# Efficacy of OSTAP, ESP block, trocar site local anesthetic injection in elective laparoscopic cholecystectomy: A randomized controlled trial

**DOI:** 10.1097/MD.0000000000043607

**Published:** 2025-08-01

**Authors:** Burak Nalbant, Asli Donmez, Savas Altinsoy, Fatma Kavak Akelma

**Affiliations:** a Department of Anesthesiology, Ankara Yildirim Beyazit University, Ankara, Turkey; b Department of Anesthesiology, Ankara Bilkent City Hospital, Ankara, Turkey; c Department of Anesthesiology, University of Health Science, Ankara, Turkey; d Department of Anesthesiology, Ankara Etlik City Hospital, Ankara, Turkey.

**Keywords:** cholecystectomy, postoperative pain, regional anesthesia, truncal nerve blocks

## Abstract

**Background:**

Effective postoperative pain control remains a clinical challenge in laparoscopic cholecystectomy (LC). Regional anesthesia techniques such as the erector spinae plane (ESP) block and the oblique subcostal transversus abdominis plane (OSTAP) block have gained popularity as potential alternatives to traditional analgesic methods. This double-blind, randomized controlled trial aimed to compare the analgesic efficacy of ESP block, OSTAP block, and trocar site local anesthetic (LA) infiltration in terms of postoperative pain scores and opioid consumption in patients undergoing LC.

**Methods:**

A total of 100 patients were equally randomized into 4 groups: ESP block (Group E), OSTAP block (Group T), trocar site LA infiltration (Group L), and a control group (Group C) receiving multimodal analgesia. ESP and OSTAP blocks were applied unilaterally prior to surgery. All patients were followed for 24 hours postoperatively. Pain intensity was evaluated using the visual analog scale (VAS), and cumulative tramadol consumption, additional analgesic requirements, nausea-vomiting scores, and incidence of shoulder pain were recorded. Data analysis was performed using SPSS, with Bonferroni correction applied for multiple comparisons.

**Results:**

VAS scores at all-time points were significantly lower in Groups E and L compared to Group C (*P* < .05). Although Group T also showed lower scores than Group C, this difference was not statistically significant after correction. Total tramadol consumption was significantly lower in Group E (112.2 ± 105.4 mg) and Group T (163.6 ± 127.1 mg) compared to Group C (281.8 ± 144.8 mg) and Group L (291.8 ± 131.6 mg; *P* < .001). Additional analgesic requirement rates were significantly lower in Groups E, T, and L than in Group C (*P* < .05). No adverse events such as LA toxicity or allergic reactions were observed. Nausea-vomiting scores and shoulder pain incidence were comparable across all groups.

**Conclusion:**

Both ESP and OSTAP blocks effectively reduced postoperative opioid requirements, with the ESP block demonstrating greater efficacy in terms of pain control. These findings support the use of unilateral ESP and OSTAP blocks as effective components of multimodal analgesia protocols in LC.

## 1. Introduction

Laparoscopic cholecystectomy (LC) is one of the most commonly performed surgical procedures in adults. Postoperative pain following LC arises due to tissue damage, residual pneumoperitoneum, and diaphragmatic irritation.^[1]^ The pain is typically somatic due to trocar incisions and visceral due to pneumoperitoneum, necessitating multimodal approaches targeting both components. Current strategies for postoperative analgesia in LC include systemic medications (e.g., paracetamol, NSAIDs, intravenous opioids) and various regional techniques such as epidural, paravertebral, transversus abdominis plane (TAP), and erector spinae plane (ESP) blocks.^[2]^

The ESP block, first described by Forero et al in 2016 for thoracic pain, has since shown promise in abdominal surgeries when applied at lower thoracic or lumbar levels.^[3]^ Similarly, the oblique subcostal transversus abdominis plane (OSTAP) block, a modified version of the TAP block introduced by Hebbard et al, has been effectively utilized in upper abdominal surgeries, including LC.^[4]^ Although several studies have individually assessed the efficacy of ESP or OSTAP blocks, comparative evidence regarding their relative effectiveness in LC – especially in contrast to conventional trocar site local anesthetic (LA) infiltration – is limited. Moreover, most previous studies have focused primarily on pain scores, with fewer addressing opioid consumption as a primary outcome.^[5–7]^ To address this gap, the present study compares unilateral ESP block, OSTAP block, and trocar site LA infiltration in terms of postoperative opioid (tramadol) consumption and pain scores in patients undergoing LC.

We hypothesized that both ESP and OSTAP blocks would result in significantly lower postoperative opioid consumption and pain scores compared to local anesthetic infiltration and control treatment. Our secondary aim is to compare the values of the patients’ pain scores with the incidence of possible side effect.

## 2. Methods

### 2.1. Ethics approval and trial registration

Ethics committee approval and written permission (T.C. Health Science University Diskapi Yildirim Beyazit Education and Research Hospital Ethical Committee, ref no 61/16, 25.03.2019) was obtained for this prospective randomized controlled study and was registered on clinicaltrials.gov with NCT03719157 trial number (https://clinicaltrials.gov/ct2/show/NCT03719157 – October 23, 2018). First registration date is October 25, 2018. First patient enrollment and actual study start day is April 1, 2019 and primary completion day is September 1 2019. Written informed consent was obtained from all patients.

### 2.2. Study design and blinding

This study was conducted in a double-blind manner. All patients were blinded to group allocation. Regional anesthesia procedures (ESP, OSTAP, or LA infiltration) were performed by a single experienced anesthesiologist who was not involved in postoperative data collection. Pain scores and other outcome measures were assessed by an independent investigator who was unaware of the group assignments. Randomization codes were kept in sealed envelopes and were only accessible to the anesthesiologist responsible for the block procedures.

### 2.3. Patient enrollment and group allocation

The study included patients with ASA score I–II, aged 18–65 years who would undergo elective laparoscopic cholecystectomy. Patients with a body mass index (BMI) of > 40 kg/m^2^, an ASA score of > III, with previous abdominal surgery, pregnant or breastfeeding patients, those with coagulopathy, known LA allergy, or infection at the injection site were excluded from the study. Moreover, patients with peripheral nerve disease, without the ability to use the PCA or visual analog scale (VAS) assessment, or those with no written consent or who were switched to open surgery were excluded from the study.

The patients were divided into 4 groups by computer-assisted randomization as ESP block group (Group E), oblique subcostal TAP (OSTAP) block group (Group T), local anesthetic infiltration group (Group L), and control group (Group C). Midazolam 0.05 mg/kg IV was administered to all patients for premedication.

### 2.4. Anesthesia and surgical procedure

After the patients were taken to the operating table, the standard ASA monitoring (electrocardiogram, noninvasive blood pressure, O_2_ saturation, and body temperature) was performed. General anesthesia was induced with 1 µg/kg fentanyl, 2–3 mg/kg propofol, and 0.6 mg/kg rocuronium bromide in all patients. 50%–50% O_2_-air, sevoflurane at 0.8 minimum alveolar concentration, and 0.01–0.2 µg/kg/min remifentanil were used for anesthesia maintenance. The remifentanil dose range was adjusted according to the patient’s hemodynamic status. At the end of the surgery, pneumoperitoneum was evacuated and the patients were extubated and taken to the postoperative recovery room.

To prevent postoperative nausea and vomiting, 4 mg ondansetron was intravenously administered during bleeding control to all patients. 10 mg metoclopramide HCl was administered intravenously to patients if they complained of nausea and vomiting (PONV) in the postoperative period.

### 2.5. ESP block procedure

A unilateral ESP block was applied to Group E at the T8 vertebra level in the lateral position after the patient was intubated by a blinded researcher during the data collection phase. A high-frequency linear probe was placed 2–3 cm lateral to the spinous process and the erector spinae plane on the transverse process was found to be contracted. The needle was advanced in the craniocaudal direction using the in-plane technique. 20 mL of LA (15 mL of 0.5% bupivacaine and 5 mL 2% lidocaine) was injected into the targeted area. The cranial and caudal spread of the LA was observed.

### 2.6. OSTAP block procedure

The OSTAP block was applied unilaterally to Group T as described by Hebbard^[8]^ after the patient was intubated following the induction of general anesthesia. In patients in the supine position, the USG probe was placed parallel to the right costal border and obliquely to the sagittal plane from the end of the xiphoid process of the sternum. A scan was performed to view the rectus abdominis muscles, posterior rectus sheath, and transversus abdominis muscles deep inside the posterior rectus sheath together. After the current image was obtained, the area between the rectus sheath and the fascia of the transversus abdominis muscle was targeted. The target was reached by inserting the peripheral block needle parallel to the USG probe from the side of the xiphoid protrusion and moving it towards the inferolateral with the “in-plane” method. Local anesthetic (15 mL of 0.5% bupivacaine and 5 mL 2% lidocaine) was injected and its distribution was monitored by USG. It was observed that the transversus abdominis muscle was pushed posteriorly during the injection.

### 2.7. Local anesthetic infiltration

Following the infiltration rules, LA (15 mL of 0.5% bupivacaine and 5 mL 2% lidocaine) was applied to the trocar sites of skin, fascia, muscle, and preperitoneal area before the trocar placement procedure by the surgical team during the operation. A total of 20 mL of LA was used, with 6 mL for trocar sites of 10 mm, and 4 mL for trocar sites of 5 mm.^[9]^ No intervention was made in the control group.

### 2.8. Postoperative management

For postoperative pain management, patients were administered perioperatively 1 g of paracetamol and 50 mg of dexketoprofen after induction of anesthesia. In the postoperative period, tramadol PCA (20 mg bolus dose -20 minutes lock) device (CADD-Legacy® PCA pump, Smiths Medical, USA) was used. The total tramadol consumption amounts were recorded at 0 (T1), 2 (T2), 4 (T3), 8 (T4), 12 (T5), and 24 (T6) hours. If the patients complained of pain during their follow-up (VAS > 4), 75 mg diclofenac sodium was intramuscularly administered as a rescue analgesic and recorded as an additional analgesic requirement. Patients with shoulder pain were also recorded.

Postoperative pain assessment was recorded at 0 (T1), 2 (T2), 4 (T3), 8 (T4), 12 (T5), and 24 (T6) hours both in motion and at rest using VAS. The patients were asked to specify the value of pain severity they felt at that time on a scale where 0-point corresponds to a pain-free condition, and 10 points correspond to the most severe pain in their lives. Nausea and vomiting statuses were monitored starting from the time of being taken to the postoperative recovery room. Nausea-vomiting severity was evaluated and recorded using a 4-point Likert scale (0: None, 1: nausea, 2: gagging, 3: vomiting). Postoperative patient follow-up and outcome assessment were conducted by a blinded investigator who was unaware of the patient’s group allocation.

### 2.9. Statistical analysis method

While calculating the sample size, the first 10 patients were considered as a pilot study because there was no study in the literature in which unilateral block was applied. It was calculated that there should be 20 patients in each group, taking the α error as 0.05 and the working power as 90% according to their 24-hour tramadol consumption. Expecting that there could be patients that might be excluded from the study, 30 patients were included in each group. Data analysis was performed using IBM SPSS Statistics version 17.0 software (IBM Corporation, Armonk, NY, USA). Whether the distributions of continuous variables were normally or not being determined Kolmogorov-Smirnov test. The assumption of homogeneity of variances was investigated Levene test. Categorical data were expressed as numbers (n) and percentage (%) while quantitative data were given as mean ± SD and median (25th–75th) percentiles. While the mean differences among groups were compared One-Way ANOVA, otherwise Kruskal–Wallis test applied for the comparisons of the quantitative data which the parametrical test assumptions were not met. Whether the differences in resting VAS, movement VAS, nausea and vomiting scores among follow-up times within each group were statistically significant or not were analyzed Friedman test. When the p-values from Kruskal–Wallis or Friedman tests were statistically significant, Dunn-Bonferroni test was used to know which group or follow-up time differ from which others. Pearson Chi-square test was used in the analysis of categorical data unless otherwise stated. On the other hand, in all 2 × 2 contingency tables to compare categorical variables; the continuity corrected Chi-square test was used when one or more of the cells had an expected frequency of 5 to 25, otherwise, the Fisher exact test was used when one or more of the cells had an expected frequency of 5 or less. In all R × C contingency tables to compare categorical variables; the likelihood ratio test was used when ¼ or more of the cells had an expected frequency of 5 or less. *P* < .05 was considered statistically significant. However, for all possible multiple comparisons the Bonferroni correction was applied for controlling Type I error.

## 3. Results

### 3.1. Patient enrollment and baseline characteristics

**Figure 1.**
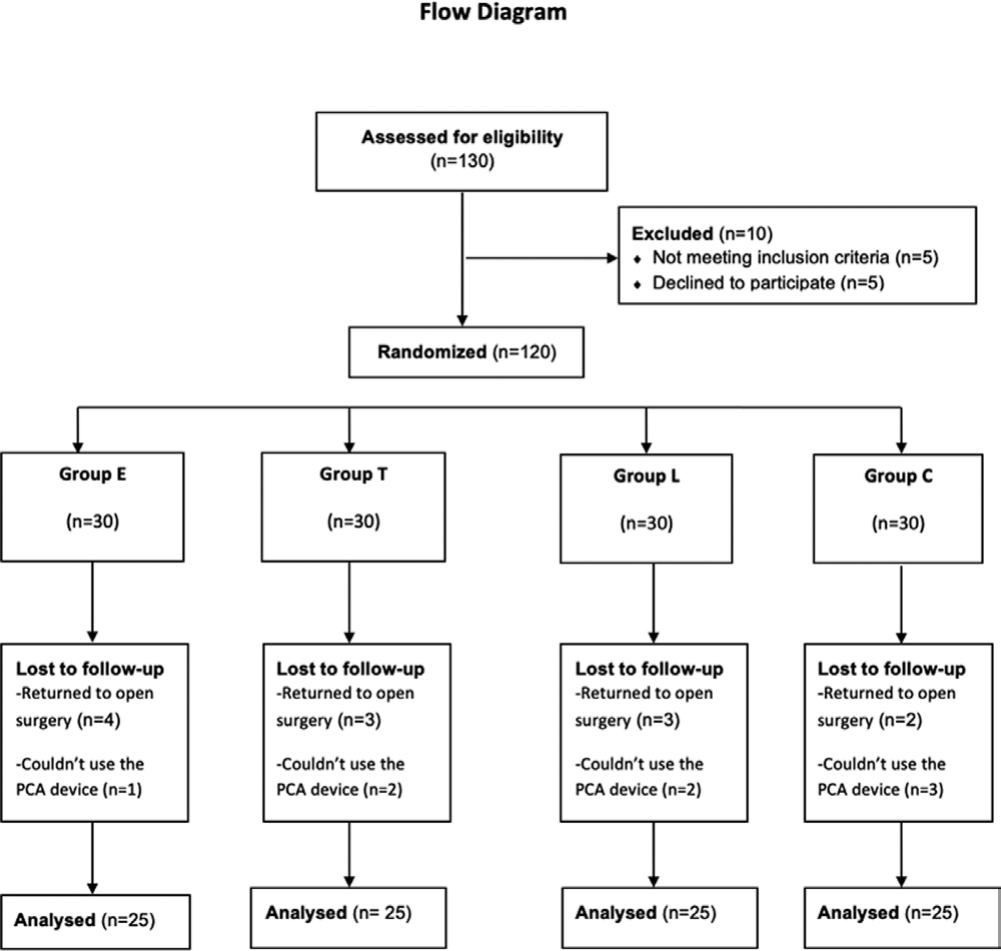
Study flow diagram.

One hundred thirty patients were enrolled to study. Five patients declined to participate and 5 patients didn’t meet inclusion criteria and were excluded from study. Four patients in Group E, 3 patients in Group T, 2 patients in Group C, and 3 patients in Group L were returned to open surgery and excluded. One patient in Group E, 2 patients in Group T, 3 patients in Group C and 2 patients in Group L couldn’t use the PCA device so they were excluded from study. Finally, 100 patients were analyzed (Fig. 1). Demographic variables and clinical characteristics of the patients were similar in all groups (Table 1).

**Table 1:** Demographic, anthropometric and clinical characteristics of the patients.

	Group C (n = 25)	Group T (n = 25)	Group E (n = 25)	Group L (n = 25)	*P*-value
Age (yr) (mean ± sd)	43.8 ± 10.1	46.0 ± 9.9	46.4 ± 8.8	43.6 ± 9.2	.620
Gender (n, %) Male/female	6 (24)/19 (76)	12 (48)/13 (52)	8 (32)/17 (68)	9 (36)/16 (64)	.348
BMI (kg/m^2^) (mean ± sd)	28.2 ± 4.7	27.9 ± 3.6	27.3 ± 3.2	27.2 ± 3.2	.765
ASA (n, %) I/II	6 (24)/19 (76)	6 (24)/19 (76)	4 (16)/21 (84)	7 (28)/18 (72)	.784
Duration of surgery (min) (median, min–max)	52 (46.5–59)	52 (48–60)	50 (46–56)	48 (44–54)	.142

ASA = American Society of Anesthesiologists, BMI = body mass index, SD = standard deviation.

### 3.2. Postoperative tramadol consumption

When compared in terms of postoperative tramadol consumption, there was a significant difference among the groups starting from the T3 timeframe. In the T3 period, Group C had the highest consumption (106.8 ± 68.3 mg), significantly greater than both Group T (58.4 ± 35.9 mg; *P* < .05) and Group E (60.0 ± 54.9 mg; *P* < .05) (Table 2). This trend continued in the T4 timeframe, with consumption in Group C (166.2 ± 96.9 mg) being significantly higher than in Groups T (90.6 ± 71.9 mg; *P* < .05) and E (82.6 ± 79.5 mg; *P* < .05), while Group L (136.0 ± 63.7 mg) also showed significantly higher values than Group E (*P* < .05). In the T5 period, Group E exhibited the lowest consumption (95.4 ± 95.0 mg), significantly less than Group C (225.2 ± 118.2 mg; *P* < .05) and Group L (198.6 ± 87.9 mg; *P* < .05). By the T6 timeframe, cumulative tramadol consumption remained highest in Group C (281.8 ± 144.8 mg) and Group L (291.8 ± 131.6 mg), while Group T and Group E demonstrated significantly lower consumption (163.6 ± 127.1 mg and 112.2 ± 105.4 mg, respectively; *P* < .001) (Table 2).

**Table 2:** Tramadol consumption amounts of the cases by groups and follow-up times.

	Group C	Group T	Group E	Group L	*P*-value*
T2 (mean ± sd)	61.8 ± 48.1	35.0 ± 21.1	44.0 ± 43.8	41.0 ± 24.3	.161
T3 (mean ± sd)	106.8 ± 68.3^A,B^	58.4 ± 35.9^A^	60.0 ± 54.9^B^	82.0 ± 38.8	**.009**
T4 (mean ± sd)	166.2 ± 96.9^A,B^	90.6 ± 71.9^A^	82.6 ± 79.5^B,C^	136.0 ± 63.7^C^	**<.001**
T5 (mean ± sd)	225.2 ± 118.2^A,B^	122.8 ± 100.7^A,D^	95.4 ± 95.0^B,C^	198.6 ± 87.9^C,D^	**<.001**
T6 (mean ± sd)	281.8 ± 144.8^A,B^	163.6 ± 127.1^A,D^	112.2 ± 105.4^B,C^	291.8 ± 131.6^C,D^	**<.001**

Bold values indicate statistically significant differences (*P* < .05) between groups at each time point.

SD = standard deviation.

Superscript letters (A, B, C, D) denote specific intergroup comparisons as follows: A: Group C versus group T (*P* < .05), B: Group C versus group E (*P* < .05), C: Group E versus group L (*P* < .05), D: Group T versus group L (*P* < .05).

*Kruskal–Wallis test.

### 3.3. Additional analgesic requirement

**Figure 2.**
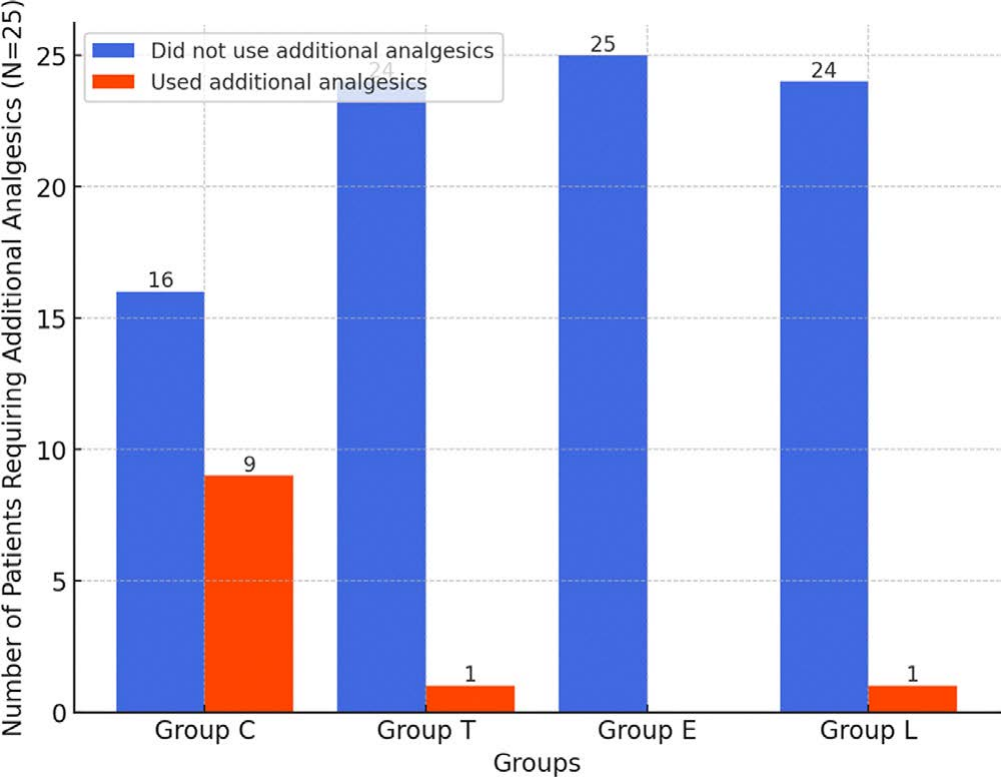
Additional analgesics requirement.

When all groups are compared in terms of additional analgesic consumption, it was observed that the need of an additional analgesic was lower in groups T, E, and L compared to Group C (*P* = .013; *P* = .002; and *P* = .013) (Fig. 2).

### 3.4. Resting pain scores (VAS)

When the groups were compared in terms of resting VAS scores, significant differences were observed at T1, T2, T3, T4, and T6 timeframes (*P* < .05) (Table 3). At T1, Group C had the highest median VAS score [5 (4.5–6)], which was significantly higher than Group E [4 (2.5–4.5); *P* < .0083] and Group L [3 (2–4.5); *P* < .001]. At T2, the resting VAS score was significantly lower in Group L [2 (1–3.5)] compared to Group C [4 (2.5–5); *P* < .001]. In the T3 timeframe, Group E [1 (0–2)] and Group L [1 (0–3)] had significantly lower VAS scores than Group C [3 (2–4); *P* < .0083]. At T6, although overall VAS scores decreased in all groups, Group T showed a slight increase [2 (0–3.5)], which was significantly higher than Group C [0 (0–1); *P* = .002] and Group E [0 (0–2); *P* = .005] (Table 3).

**Table 3:** The resting VAS levels of the cases by groups and follow-up times.

	Group C	Group T	Group E	Group L	*P*-value*
T1	5 (4.5–6)^A,B^	5 (4–5)	4 (2.5–4.5)^A^	3 (2–4.5)^B^	**<.001**
T2	4 (2.5–5)^B^	3 (2–4)	3 (1–4)	2 (1–3.5)^B^	**<.001**
T3	3 (2–4)^A,a^	2 (1–2)^a^	1 (0-2)^A,a^	1 (0–3)	**<.001**
T4	2 (1–3)^a^	1 (0–2)^a^	1 (0–1)^a^	1 (0–2.5)^a^	.028
T5	1 (0–2)^a,b^	1 (0–2)^a,b^	0 (0–1.5)^a^	1 (0–2)^a^	.600
T6	0 (0–1)^C,a,b^	2 (0–3.5)^C,D,a^	0 (0-2)^D,a^	2 (0.5–2)^a^	**.003**
*P*-value**	**<.001**	**<.001**	**<.001**	**<.001**	

The data were expressed as median (1st quartile-3rd quartile).

Bold values indicate statistically significant results.

Uppercase letters indicate significant intergroup differences; lowercase letters indicate significant intragroup differences.

VAS = Visual Analog Scale.

A: The difference between group C and group E (*P* < .0083), B: The difference between group C and group L (*P* < .001), C: The difference between group C and group T (*P* = .002), D: The difference between group T and group E (*P* = .005).

a: The difference with hour 0 (*P* < .01), b: The difference with the second hour (*P* < .001).

* *P*-values for intergroup comparisons at each follow-up time point using the Kruskal–Wallis test with Bonferroni correction (*P* < .0083 considered significant).

** *P*-values for intragroup (within-group) comparisons across time points using the Friedman test with Bonferroni correction (*P* < .0125 considered significant).

### 3.5. Movement pain scores (VAS)

When the groups were compared in terms of postoperative movement VAS scores, statistically significant differences were observed at T1, T2, T3, T4, and T6 timeframes (*P* < .05). At T1, Group C [5 (5–6)] and Group T [5 (4–6)] had significantly higher scores than Group L [3 (2–4.5); *P* < .0083 for both]. Similarly, at T2, Group C [4 (3–5)] had higher scores than Group L [2 (1–3.5); *P* < .0083]. In the T3 timeframe, both Group E [2 (0–3)] and Group L [2 (0–3)] had significantly lower scores than Group C [3 (2.5–4); *P* < .001] and Group T [2 (2–3)]. By T6, Group T had the highest movement VAS score [3 (2–4)], significantly greater than both Group C [0 (0–1.5); *P* < .001] and Group E [0 (0–2.5); *P* < .001] (Table 4).

**Table 4:** The movement VAS levels of the cases by groups and follow-up times.

	Group C	Group T	Group E	Group L	*P*-value*
T1	5 (5–6)^A^	5 (4–6)^B^	4 (3–5)	3 (2–4.5)^A,B^	**<.001**
T2	4 (3–5)^A^	4 (2.5–4)	3 (1–4.5)	2 (1–3,5)^A^	**<.001**
T3	3 (2.5–4)^A,C,a^	2 (2-3)^a^	2 (0–3)^C,a^	2 (0–3)^A^	**<.001**
T4	2 (1.5–3.5)^a^	2 (1–3)^a^	1 (0–2.5)^a^	2 (0–3)	.049
T5	1 (0.5–2.5)^a,b^	2 (1–3)^a^	1 (0-2)^a^	2 (0–3)^a^	.284
T6	0 (0–1.5)^D,a,b,c^	3 (2–4)^D,E,a^	0 (0–2.5)^E,a,b^	2 (1–2)^a^	**<.001**
*P*-value**	**<.001**	**<.001**	**<.001**	**<.001**	

The data were expressed as median (1st quartile-3rd quartile).

Bold values indicate statistically significant results.

Uppercase letters indicate significant intergroup differences; lowercase letters indicate significant intragroup differences.

VAS = Visual Analog Scale.

A: The difference between group C and group L (*P* < .0083), B: The difference between group T and group L (P < .0083), C: The difference between group C and group E (*P* < .001), D: The difference between group C and group T (*P* < .001), E: The difference between group T and group E (*P* < .001).a: The difference with hour 0 (*P* < .01), b: The difference with the 2nd hour (*P* < .01), c: The difference with the 4th hour (*P* = .007).

* *P*-values for intergroup comparisons at each follow-up time point using the Kruskal–Wallis test with Bonferroni correction (*P* < .0083 considered significant).

** *P*-values for intragroup (within-group) comparisons across time points using the Friedman test with Bonferroni correction (*P* < .0125 considered significant).

### 3.6. Postoperative adverse effects, nausea-vomiting, and shoulder pain

No adverse effects (local anesthetic toxicity (LAST), vascular injection, and allergic reaction) were encountered in any of the groups. When the groups were compared in terms of PONV, there was no difference in the T1, T2, T5, and T6 periods (*P* > .05). There was a difference between the groups in terms of PONV in the T3 and T4 timeframes (*P* < .001). It was observed that this difference was because more patients in Group L complained of nausea and vomiting in the T3 timeframe compared to Group T and Group E (*P* < .001 and *P* < .001), and in the T4 timeframe compared to Group E (*P* < .001) (Table 5). No statistically significant difference was found between the groups in terms of the frequency of shoulder pain (*P* = .753).

**Table 5:** Nausea and vomiting scores of the cases by groups and follow-up times.

	Group C	Group T	Group E	Group L	*P*-value*
T1	0 (0–1)	0 (0–0)	0 (0–1)	0 (0–0)	.506
T2	0 (0–0)	0 (0–0,5)	0 (0–0)	0 (0–0)	.371
T3	0 (0–0)	0 (0–0)^A^	0 (0–0)^B^	0 (0–1)^A,B^	**<.001**
T4	0 (0–0.5)	0 (0–0)	0 (0–0)^B^	1 (0–1)^B^	**.002**
T5	0 (0–1)	0 (0–0,5)	0 (0–0)	0 (0–0)	.445
T6	0 (0–0)	0 (0–0)	0 (0–0)	0 (0–1)	.319
*P*-value†	.637	.326	.092	.074	

The data were expressed in median (1st quartile–3rd quartile).

Bold values indicate statistically significant results.

Superscript letters A and B denote specific intergroup comparisons as follows:A: Group T versus group L (*P* < .001), B: Group E versus group L (*P* < .001).

* *P*-values for intergroup comparisons at each follow-up time point using the Kruskal–Wallis test with Bonferroni correction; *P* < .0125 was considered statistically significant.

† *P*-values for intragroup (within-group) comparisons across time points using the Friedman test with Bonferroni correction; *P* < .0125 was considered statistically significant.

## 4. Discussion

This study showed that postoperative tramadol consumption was lower in the ESP and OSTAP blocks compared to the trocar site local anesthetic infiltration, and the control groups thus, these blocks were found to provide more effective analgesia in LC.

The pain seen after LC occurs in 2 forms: somatic (parietal) fibers (skin-borne) and visceral fibers (due to peritoneal irritation). Parietal type pain is a sudden-onset, well localized, and sharp pain.^[10]^ The administration of bupivacaine on the trocar site for this purpose has been shown to prevent postoperative pain, reduce pain scores, opioid consumption, and the need for an additional analgesic after LC.^[9,11]^ In our study, 20 mL of 0.5% bupivacaine was administered intraoperatively in Group L, similar to the literature. Likewise, it reduced analgesic consumption, pain scores, and additional analgesic needs compared to the control group.^[2,9,12]^

The TAP block has been widely used in LC. However, in recent years, it has been reported that the subcostal approach provides better analgesic efficacy and lower VAS scores.^[7,13–15]^ Oksar et al, comparing the OSTAP technique with the classical TAP and the control group in LC, found that postoperative pain scores were lower in the OSTAP group. In terms of tramadol consumption, OSTAP and TAP were similar but had lower values than the control group. In our study, we chose the OSTAP technique with proven efficiency in the application of the TAP block and found that tramadol consumption was lower in Group T than in the control group at all timeframes except for T2. In another study, both VAS values and tramadol consumption were found to be lower in OSTAP group than the control group.^[16]^ On the contrary, as a result of the study performed by Houben et al showed that VAS values and additional analgesic needs were similar in subcostal TAP block and the control group. They stated that this was because the analgesic effect of the TAP block was immeasurably low when adequate multimodal analgesia was provided to the control group.^[17]^ In this study, while there was no difference in terms of resting and movement VAS values in all the time frames except T6 between Group T and the control group, lower VAS values were observed in the control group in the T6 timeframe. We think that this is due to the high tramadol consumption in the control group, similar to the literature.

Suseela et al compared the OSTAP block with trocar site infiltration and showed that the OSTAP block group had lower VAS scores, tramadol, and additional analgesic consumption.^[18]^ In current study VAS values were lower in Group L than Group T in T1 and T2. In the other periods there was no significant difference. In terms of tramadol consumption, higher tramadol consumption was observed in Group L compared to Group T in all timeframes. The low tramadol consumption values of the OSTAP group show us that the OSTAP block reduced opioid agent consumption by providing a longer and more effective analgesic method than local anesthetic infiltration. We think that the different duration of TAP block and infiltration anesthesia using the same agent can be explained by the transversus abdominis plan has a less dense vascular network compared to the skin and subcutaneous tissues, and accordingly shows slower LA absorption.

The ESP block is a regional anesthesia method to prevent thoracic and abdominal pain.^[2,3]^ Differing theories have been put forward on the action mechanism of the ESP block. In a cadaveric study ESP block by administering 20 mL of contrast material at the T5 level, it was thought that the contrast agent created an analgesic effect by reaching the paravertebral area and thoracic spinal nerves through the superior costotransverse ligament.^[19]^ In another cadaver study, 20 mL of methylene blue was spread to the paravertebral, intercostal, and, in some cases, the prevertebral area.^[20]^ The magnetic resonance imaging study by Schwartzmann et al, showed that the contrast material involvement extended to the paravertebral, epidural, and intervertebral foramen in the T5–T12 range after injection using a volume of 30 mL from the T10 level. They also found that the contrast material in the unilateral ESP block passed to the contralateral area and held the epidural space circumferentially. This study suggests that the ESP block reduces both visceral and somatic pain due to transforaminal and epidural spread.^[21]^ Tulgar et al also found a bilateral sensory block after an unilateral ESP block from the T9 level.^[22]^ All these studies examining the effect of the ESP block have made us think that ESP can provide analgesic efficacy when done unilaterally. However, its concentration and volume are among the most important risk factors in the LAST, which is one of the most feared complications in regional anesthesia applications with the highest mortality rates.^[23]^ For these reasons, we reduced the dose and volume of the LA by unilaterally applying the ESP and OSTAP blocks, whose effect was shown in our study when applied unilaterally.

Tulgar et al showed that additional analgesic need and tramadol consumption decreased significantly in the ESP group in LC.^[24]^ In a similar study, it was shown that 24-hour morphine consumption and VAS values significantly decreased with the bilateral ESP block at the T8 level.^[25]^ In our study, VAS levels (both rest and movement) and tramadol consumption were low in Group E than control group. Our findings show that the ESP block can be used effectively to reduce opioid consumption.

Altiparmak et al found that lower pain scores and lower consumption of additional analgesic and tramadol in the ESP group than OSTAP group.^[26]^ In another study, higher postoperative pain scores and analgesic consumption were observed in the control group compared to the ESP and OSTAP groups. However, when the ESP and OSTAP groups were compared, there was no difference between the 2 groups.^[12]^ In our study, VAS values and tramadol consumption were low in the ESP block group without statistically significant difference. And, additional analgesic requirements were similar. This suggests that both blocks do not have a distinct advantage over each other.

In the literature, there were no significant differences between ESP block, OSTAP block, and local anesthetic infiltration in terms of shoulder pain frequency.^[13,27]^ Similarly, in our study, the frequency of shoulder pain was similar as literature.

In our study, we routinely administered ondansetron in the intraoperative period to prevent PONV. Chen et al compared the OSTAP block and IV morphine administration in LC, and found no difference between the 2 groups in terms of PONV.^[7]^ Similarly, we used tramadol, an opioid derivative, and observed results consistent with the literature. However, we found higher PONV scores in Group L in the T3 and T4 timeframes compared to other groups. This may be due to the increased tramadol consumption in Group L after the 4th hour compared to the other groups.

As far as we know, our study is the first study in which the ESP and OSTAP blocks were performed and compared unilaterally. The fact that contralateral LA spread in ESP block has been demonstrated in studies and that effective analgesia can be achieved with unilateral OSTAP block^[14]^ led us to think that these blocks can also be effective when applied unilaterally, and guided us to conduct this study.

We believe that since lower VAS levels were achieved especially in the ESP block group, although not statistically significant, the unilateral application has positive effects in terms of both patient comfort and low dose LA use. This is because the OSTAP block can usually be easily performed under general anesthesia as it is applied in the supine position. However, since the ESP block requires a lateral decubitus or prone position, it was applied under preoperative sedation in some studies.^[25]^ Since this is a double-blind study, we could not apply the ESP block in awake patients. However, we believe that applying the ESP block to awake patients may be safer in terms of positioning and early detection of complications that may occur during the block.

Our study has some limitations. Since we did not track the patients in the postoperative period except for the first 24 hours, we were unable to evaluate the long-term effects of the methods used on pain scores and complications. Another limitation is that we did not perform a dermatomal examination to reveal the sensorial block-level. For this purpose and to guide future studies, we recommend future studies to perform dermatomal examinations for sensorial blocks in patients to examine the effectiveness of unilateral blocks.

## 5. Conclusion

As a result of our study, both the ESP and OSTAP block groups provided less additional analgesic and less tramadol consumption, and the ESP block was especially more effective in terms of VAS scores. We think that these blocks provide effective analgesia when applied unilaterally and OSTAP or ESP blocks may be preferred unilaterally for postoperative analgesia, but further studies are needed on this subject.

## Author contributions

**Conceptualization:** Burak Nalbant, Asli Donmez.

**Formal analysis:** Savas Altinsoy, Fatma Kavak Akelma.

**Investigation:** Burak Nalbant, Savas Altinsoy.

**Methodology:** Asli Donmez.

**Project administration:** Burak Nalbant, Savas Altinsoy, Fatma Kavak Akelma.

**Supervision:** Asli Donmez.

**Writing – original draft:** Burak Nalbant, Savas Altinsoy, Fatma Kavak Akelma.

**Writing – review & editing:** Burak Nalbant, Savas Altinsoy, Fatma Kavak Akelma.
